# The survivin-ran inhibitor LLP-3 decreases oxidative phosphorylation, glycolysis and growth of neuroblastoma cells

**DOI:** 10.1186/s12885-023-11635-2

**Published:** 2023-11-25

**Authors:** Celimene Galiger, Fatema Tuj Zohora, Carmen Dorneburg, Daniel Tews, Klaus-Michael Debatin, Christian Beltinger

**Affiliations:** 1 Department of Pediatrics and Adolescent Medicine, Section of Experimental Pediatric Oncology, University Medical Center Ulm, Eythstr. 24, Ulm, 89075 Germany; 2 Department of Pediatrics and Adolescent Medicine, University Medical Center Ulm, Eythstr. 24, Ulm, 89075 Germany

**Keywords:** LLP-3, Neuroblastoma, Survivin, Ran, OXPHOS, Glycolysis, HK2, HIF-1α

## Abstract

**Background:**

Neuroblastoma (NB), the most common extracranial solid malignancy in children, carries a poor prognosis in high-risk disease, thus requiring novel therapeutic approaches. Survivin is overexpressed in NB, has pro-mitotic and anti-apoptotic functions, and impacts on oxidative phosphorylation (OXPHOS) and aerobic glycolysis. The subcellular localization and hence function of survivin is directed by the GTPase Ran.

**Aim:**

To determine efficacy and modes of action of the survivin-Ran inhibitor LLP-3 as a potential novel therapy of NB.

**Methods:**

Survivin and Ran mRNA expression in NB tumors was correlated to patient survival. Response to LLP-3 in NB cell lines was determined by assays for viability, proliferation, apoptosis, clonogenicity and anchorage-independent growth. Interaction of survivin and Ran was assessed by proximity-linked ligation assay and their subcellular distribution by confocal immunofluorescence microscopy. Expression of survivin, Ran and proteins important for OXPHOS and glycolysis was determined by Western blot, hexokinase activity by enzymatic assay, interaction of survivin with HIF-1α by co-IP, and OXPHOS and glycolysis by extracellular flux analyzer.

**Results:**

High mRNA expression of survivin and Ran is correlated with poor patient survival. LLP-3 decreases viability, induces apoptosis, and inhibits clonogenic and anchorage-independent growth in NB cell lines, including those with *MYCN* amplification, and mutations of p53 and ALK. LLP-3 inhibits interaction of survivin with Ran, decreasing their concentration both in the cytoplasm and the nucleus. LLP-3 impairs flexibility of energy metabolism by inhibiting both OXPHOS and glycolysis. Metabolic inhibition is associated with mitochondrial dysfunction and attenuated hexokinase activity but is independent of HIF-1α.

**Conclusion:**

LLP-3 attenuates interaction and concentration of survivin and Ran in NB cells. It controls NB cells with diverse genetic alterations, associated with inhibition of OXPHOS, aerobic glycolysis, mitochondrial function and HK activity. Thus, LLP-3 warrants further studies as a novel drug against NB.

**Supplementary Information:**

The online version contains supplementary material available at 10.1186/s12885-023-11635-2.

## Introduction

In neuroblastoma (NB), the most common extracranial solid malignancy in children with a poor prognosis in high-risk disease, gain of 17q, where *BIRC5* (*survivin*) resides, is common [1, 2]. NB was one of the first cancers where survivin was found to play a pivotal role in aggressiveness [3]. In addition to its cytoplasmic anti-apoptotic function, nuclear survivin is important for mitosis in cancers [4], including NB.

Survivin is shuttled between the nucleus and the cytoplasm with help of the GTPase Ran. This is mediated by the nuclear export signal (NES) of survivin interacting with exportin1 (Crm1) in the presence of Ran-GTP [5]. While Ran is known to promote tumorigenesis of NB within a LIN28B-Ran-AURKA signaling network [6], neither its impact on prognosis nor its subcellular localization and potential interaction with survivin have been investigated yet in NB.

More recently, a pool of survivin has been described to be present in the mitochondrial membrane of cancer cells, including NB, but not of normal cells [7–9]. There is clear evidence that mitochondrial survivin impacts on OXPHOS and aerobic glycolysis [7–9], albeit the nature of this impact remains controversial [4]. On the one hand, survivin has been reported to enhance OXPHOS by stabilizing OXPHOS Complex II [8]. On the other hand, survivin has been shown to reduce OXPHOS by inducing mitochondrial fragmentation and inhibiting Complex I, concomitantly enhancing aerobic glycolysis [7]. Along this line, survivin reduced OXPHOS by inhibition of mitophagy leading to accumulation of defective mitochondria, thus increasing dependency on glycolysis [9].

Given the importance of survivin in maintaining cancers, survivin inhibitors have been developed. YM155, a transcription inhibitor thought to specifically inhibit transcription of survivin, showed preclinical efficacy in several cancers, including NB [10], but had limited success in clinical trials [4]. Decreasing expression of survivin by 2-deoxy-glucose inhibited NB cells [11]. Small molecules disrupting survivin homo- or heterodimers, such as sheperdin [12], S12 [13], LQZ-7F [14] or LLP-3 [15–17] are still in the preclinical state of assessment. LLP-3, a promising candidate, binds near the dimer interface of survivin disrupting its interaction with Ran, thus impairing glioma stem cell survival and growth in vitro and in vivo [15].

We set out to determine efficacy and modes of action of the survivin inhibitor LLP-3 as a potential novel therapy of NB. This paper shows that LLP-3 is effective against NB cells by decreasing interaction and levels of survivin and Ran, and impairing flexibility of energy metabolism.

## Material and methods

### Association of Birc5 and Ran mRNA expression in NB with prognosis

Clinically annotated Birc5 and Ran transcript levels previously determined by mRNA sequencing in 498 NB (GSE62564) were employed for in silico analysis using the R2 Genomics Analysis and Visualization Platform (http://r2.amc.nl).

### Chemicals

LLP-3 (SML0991), IGEPAL (NP-40, I8896), cobalt (II) chloride (203,084), 3-(4,5-dimethylthiazol-2-yl)-2,5-diphenyltetrazolium (MTT, M2128), Triton X-100 (T8787), and propidium iodide (P4864) were from Sigma-Aldrich. Cycloheximide (ALX-380–269-G005) was acquired from ENZO Life Sciences and sodium citrate (A2403) from AppliChem.

### Cell culture

The human NB cell lines SK-N-AS and SK-N-BE(2)-C were purchased from ATCC (American Type Culture Collection), GI-M-EN, SH-SY5Y, IMR32, LAN5 and KELLY cells from DSMZ (German Collection of Microorganisms and Cell Cultures), NB69 from ECACC (European Collection of Authenticated Cell Cultures), and IMR5 and NLF cells were a gift from G. M. Brodeur (CHOP, Philadelphia, PA). SK-N-AS cells were cultured in Dulbecco’s minimum essential medium (DMEM; Gibco) supplemented with 10% of heat inactivated fetal calf serum (FCS; Gibco), SH-SY5Y cells in DMEM with 20% FCS, SK-N-BE(2)-C cells in a 1:1 mixture of DMEM and Ham’s F12 (Gibco) with 10% FCS, GI-M-EN and KELLY cells in RPMI 1640 medium (Gibco) with 10% FCS, NB69 in RPMI 1640 medium with 15% FCS, and IMR5, NLF, IMR32 and LAN5 cells in RPMI 1640 medium with 20% FCS. All media were supplemented with 2 mM L-glutamine (Gibco) and 100 U/ml penicillin/streptomycin (Gibco) and are henceforth called growth media. All cell lines were negative for mycoplasma. Short-tandem repeat profiling was performed to confirm the identity of the cells.

### Cell proliferation analysis by dye dilution

Cells were labelled using the Cell Trace Violet-Cell Proliferation Kit (C34557, Thermo Fisher Scientific) according to the manufacturer's instructions. Cells were harvested, washed once with PBS and 1×10^6^ cells resuspended in PBS were mixed 1:1 with the working dye solution (2–4 μM in PBS) for 20 min at 37 °C/5% CO_2_, protected from light. 500 µl of FCS was added and incubated for 5 min at 37 °C to remove free dye. Cells were washed with PBS. 2×10^4^ labelled cells were seeded per well in 12-well plates, allowed to attach overnight and incubated with increasing concentrations of LLP-3. 24 h after start of treatment, mean fluorescence intensity was quantified daily for 5 days by flow cytometry (Attune NxT Cytometer) and the data were analyzed using FlowJo v10 software.

### MTT assay

1×10^4^ cells were plated in 96-well plates and incubated overnight. The following day, increasing concentrations of LLP-3 were added for 72 h. Cell viability was measured by MTT assay, with the viability of DMSO-treated controls set at 100%. IC_50_ values were calculated using GraphPad Prism version 8 (GraphPad).

### Clonogenic growth assay

0.75×10^3^ cells per well were seeded into 6-well plates and allowed to attach overnight in 2 ml growth medium and treated with LLP 3 on days 1 and 3. Colonies were stained with crystal violet solution in 3.7% formaldehyde.

### Soft agar growth assay

Experiments were carried out in 24-well plates with a layer of 0.6% agar in growth medium. 2×10^3^ cells per well were seeded as single-cell suspension in 0.5% agar. LLP-3 in 1 ml of growth medium was added above the top agar on days 1 and 3. After culturing for 2 weeks (KELLY) or 3 weeks (SK-N-AS), colonies that had formed within the soft agar were stained with 1 mg/ml MTT in PBS (Gibco).

### Apoptosis assay

Late apoptosis was determined by quantification of DNA fragmentation using FACS analysis of propidium iodide-stained nuclei. Cells were resuspended in 300 µl of hypotonic fluorochrome solution containing 0.1% sodium citrate, 0.1% Triton X-100 and 50 µg/ml propidium iodide in distilled water. Cells were incubated at 4 °C overnight and late apoptosis was measured by flow cytometry.

### HK activity assay

HK activity of SK-N-AS and KELLY cells was determined using the Hexokinase Colorimetric Assay Kit (MAK091, Sigma). Briefly, 1×10^6^ cells were homogenized in 200 µL of ice-cold HK assay buffer and centrifuged at 13 000*g* for 10 min at 4° C. The supernatant was diluted 1:10 and 1:5 for SKNAS and KELLY cells, respectively, and mixed with reagents as stipulated by the manufacturer. HK activity was determined by colorimetric assay. Absorbance was measured at 450 nm wavelength using ta microplate reader (TECAN)**.**

### Co-IP and Western blot analysis

For Co-IP of survivin with Ran, cells were treated with 25 µM of LLP-3 for 4 h or were left untreated. For Co-IP of survivin with HIF-1α, cells were subjected to cobalt (II) chloride (CoCl_2_, 200 µM for KELLY cells and 400 µM for SK-N-AS cells) for 2 h to stabilize HIF-1α. Cells were lysed using a non-denaturing lysis buffer Tris–HCl, (30 mM, pH 7.4), NaCl (120 mM), EDTA (2 mM), KCl (2 mM), 10% glycerol, 1% NP-40 and Protease Inhibitor Cocktail EDTA-free (Roche). Pre-cleared lysates containing 500 µg of total protein were incubated with 4 µg rabbit anti-HIF-1α (20,960–1-AP, Proteintech), 2 µg rabbit anti-survivin (10,508–1-AP, Proteintech) and 2–4 µg rabbit IgG (2729, Cell Signaling Technology), and with 30 μl of protein A agarose beads (16–125, Merck Millipore) overnight at 4 °C. For Western blot analysis of OXPHOS and glycolysis proteins after LLP-3 treatment, cells were lysed in RIPA buffer (Tris pH8, 50 mM), NaCl (150 mM), 1% NP-40, 0.1% SDS, 1% DOC (sodium-deoxycholate), EDTA (pH8, 1 mM), 2 mM DTT (dithiothreitol) and Protease Inhibitor Cocktail EDTA-free (Roche). Protein lysates were run at 200 V in Bis–Tris 4–12% gradient Mini Protein Gels of 1.0 mm thickness (NP0321BOX, ThermoFisher) and transferred to nitrocellulose membranes. Membranes were blocked with 5% nonfat dried milk in TBS-T for 1 h at room temperature and incubated overnight at 4 °C with the following antibodies: mouse anti-survivin (66495–1-lg, Proteintech, 1:1000), rabbit anti-survivin (AF886, R&D, 1:1000), rabbit anti-Ran (10469–1-AP, Proteintech, 1:500), mouse anti-HIF-1α (610958, BD Transduction Laboratories, 1:1000), rabbit anti-GLUT1 (NB110-39113, Novus Biologicals, 1:500), rabbit anti-Hexokinase 2 (22029–1-AP, Proteintech, 1:2000), rabbit anti-LDHA (SAB1100050, Sigma, 1:500), mouse anti-PKM2 (60268–1-Ig, Proteintech, 1:5000), rabbit anti-PDK1 (10026–1-AP, Proteintech, 1:500), rabbit anti-PDHA (66119–1-Ig Proteintech, 1:1000), mouse anti-GAPDH (5G4-6C5, HyTest, 1:8000) and mouse anti-β-actin (A5441 clone AC-15, Sigma, 1:8000). As secondary antibodies, goat anti-mouse IgG (H+L)-HRP conjugate (170–6516, BioRad,1:10000) and goat anti-rabbit IgG (H+L)-HRP conjugate (65–6120, Invitrogen, 1:10000) were used for 1 h at room temperature. ECL (GE Healthcare) and WESTAR ETA ULTRA (Cyanagen) reagents were used to visualize the proteins of interest and the ChemiDoc MP Imaging System (Bio-Rad) for imaging of Western blots. Densitometry was performed using ImageJ processing software (Fiji).

### Proximity Ligation Assay (PLA)

8-well chamber slides (354118, Falcon) were pre-coated with collagen for 1.5 h and 8×10^4^ SK-N-AS and KELLY cells were seeded per well overnight to attach. Cells were treated with and without 25 µM of LLP-3 for 12 h and 24 h. Cells were fixed with PEM buffer (80 mM PIPES+5 mM EGTA+1 mM MgCl2, pH 7.4) for 15 min followed by permeabilization in PEMT buffer (PEM+0.2% Tween 20, pH 7.4) for 15 min. Cells were blocked in blocking buffer (PEMT+0.5% BSA+10% NGS) for 1 h 37 °C in a humidity chamber followed by overnight incubation with rabbit-α-Survivin polyclonal (Proteintech 105–1-AP, 1:200) and mouse-Ran monoclonal (Thermo Fisher Scientific, MA1-20581, 1:500) at 4 °C. Afterwards, probe incubation, ligation and amplification steps were performed according to the manufacturer’s recommendations (NaveniFlex MR kit, NAV-NF.MR.100). Cells were incubated with mouse FITC α-Tubulin (Abcam 64503, 1 µg/ml) and DAPI (Molecular Probes, H3570, 1:5000) for 1 h at room temperature for cytoskeletal and nuclear counterstaining, respectively. Images were taken with a Leica TCS SP8 confocal microscope using a 40×NA 1.3 lens. Two images (random visual field of 290.91×290.91 microns) per each of three independent replicates were used for quantification of PLA signals using ImageJ software (Fiji).

### Confocal microscopy

8-well chamber slides (354118, Falcon) were coated with collagen for 1.5 h and 8×10^4^ SK-N-AS and Kelly cells were seeded per well overnight to attach. Cells were treated with and without 25 µM of LLP-3 for 12 h and 24 h. Cells were fixed with PEM buffer (80 mM PIPES+5 mM EGTA+1 mM MgCl2, pH 7.4) for 15 min followed by permeabilization in PEMT buffer (PEM+0.2% Tween 20, pH 7.4) for 15 min. Cells were blocked in blocking buffer (PEMT+0.5% BSA+10% NGS) for 1 h at room temperature followed by overnight incubation with rabbit-α-survivin polyclonal (Proteintech 105–1-AP, 1:200) and mouse-Ran monoclonal (Thermo Fisher Scientific, MA1-20,581, 1:500) at 4 °C. Cells were washed with PEMT buffer before incubation with Alexa 488 goat-anti-rabbit IgG, Abcam, ab150081, 1:1000), Alexa 568 goat-anti-mouse IgG (Thermo Fisher Scientific, A-11031, 1:1000) and DAPI (Molecular Probes, H3570, 1:5000) for 1 h at room temperature. Slides were mounted with DAKO fluorescent mounting medium (Dako, S3023).

Images were taken with a Leica TCS SP8 confocal microscope using a 40×NA 1.3 lens. 1234 to 1313 and 1047 to 1316 SK-N-AS cells per condition were analyzed at 12 and 24 h of treatment, respectively. As for Kelly cells, 511 to 670 and 288 to 472 cells were investigated at those time points. Cells from two random visual fields (each measuring 290.77×290.77 microns) from three independent experiments, resulting in a total of 6 visual fields, were analyzed.

Fluorescence intensity of survivin and Ran in the nucleus and the cytoplasm of each cell was determined using ImageJ software (Fiji). As SK-N-AS tend to grow as clearly separated cells, their outline was readily visible using appropriate ImageJ contrast settings. As Kelly cells touch or overlap, a different approach was used to define their cellular outlines. Here, the entire field of view was imaged and gaps in the cell layers were masked. The remaining, cell-covered area was segmented using a Voronoi algorithm centered on the nuclei. This assigns the surrounding cytoplasm to each nucleus. For both cell lines, cytoplasmic intensities were then calculated by taking the total area of the cell or segmented unit approximating one cell, multiplying it with the overall average intensity, subtracting the product of nuclear area and nuclear intensity, and dividing the remainder by the difference of total and nuclear area.

### Time course analysis of HIF-1α

SK-N-AS and KELLY cells were treated with LLP-3 (25 µM) or were left untreated for 2, 4, 6, 8 and 12 h. At each time point, the growth medium containing LLP-3 was removed and replaced by medium containing 200 µM (KELLY) and 400 µM (SK-N-AS) CoCl_2_ for 2 h to stabilize HIF-1α. Cells were harvested and subjected to Western blot analysis using mouse anti-HIF-1α (610,958, BD Transduction Laboratories, 1:1000).

### Seahorse extracellular flux analysis

Cells were seeded in XF96 cell culture microplates (10,185–004, Agilent Technologies) at 3×10^4^ cells per well in 100 µl DMEM or RPMI growth medium supplemented with FCS and incubated at 37 °C and 5% CO_2_ overnight. Cells were treated with increasing concentrations of LLP-3 for 4 h, with or without CoCl_2_ (200 µM for KELLY cells and 400 µM for SK-N-AS cells) for 2 h to stabilize HIF-1α. Growth medium was removed and replaced with prewarmed XF assay medium (103,575–100, Agilent Technologies) containing 2 mM of L-glutamine (25,030–024, Gibco). The Seahorse XF Cell Mito Stress Kit was used for analysis (103,015–100, Agilent Technologies). Oxygen consumption and extracellular acidification rates (OCR and ECAR) were measured simultaneously using a Seahorse XFe96 Flux Analyzer (Agilent Technologies). ATP-linked as well as uncoupled (proton leak) respiration was profiled by injecting 2 μM oligomycin (inhibiting ATP synthase), and full substrate oxidation capacity was determined by injecting 0.5 μM carbonyl cyanide-4 (trifluoromethoxy) phenylhydrazone (FCCP) in combination with 1 µM pyruvate (11,360–39, Gibco). Non-mitochondrial respiration was determined by injecting 0.5 μM antimycin A and 0.5 μM rotenone (inhibiting electron flux through complex I and III). ECAR profile was generated by injecting 10 µM glucose. To determine non-glycolytic acidification, 50 mM 2-deoxy-d-glucose (D8375, Sigma) was used. OCRs and ECARs were determined by machine algorithms and plotted against time.

### Statistical analysis

In general, the average from at least three independent experiments was used. IC50s were calculated by non-linear regression. Means of two groups of cell lines with different genetic status were analyzed using the unpaired t-test. Proliferation, apoptosis, colony, metabolic assays and densitometry of treatment groups and controls were analyzed by one-way and two-way analysis of variance (ANOVA). Differences between groups were considered to be significant at a *p* value of<0.05. Statistical analysis, including the calculation of errors and *p*-values, were performed with GraphPad Prism Software version 8 (GraphPad, San Diego, CA).

## Results

### High expression of survivin and Ran conveys poor prognosis in NB

**Fig. 1:**
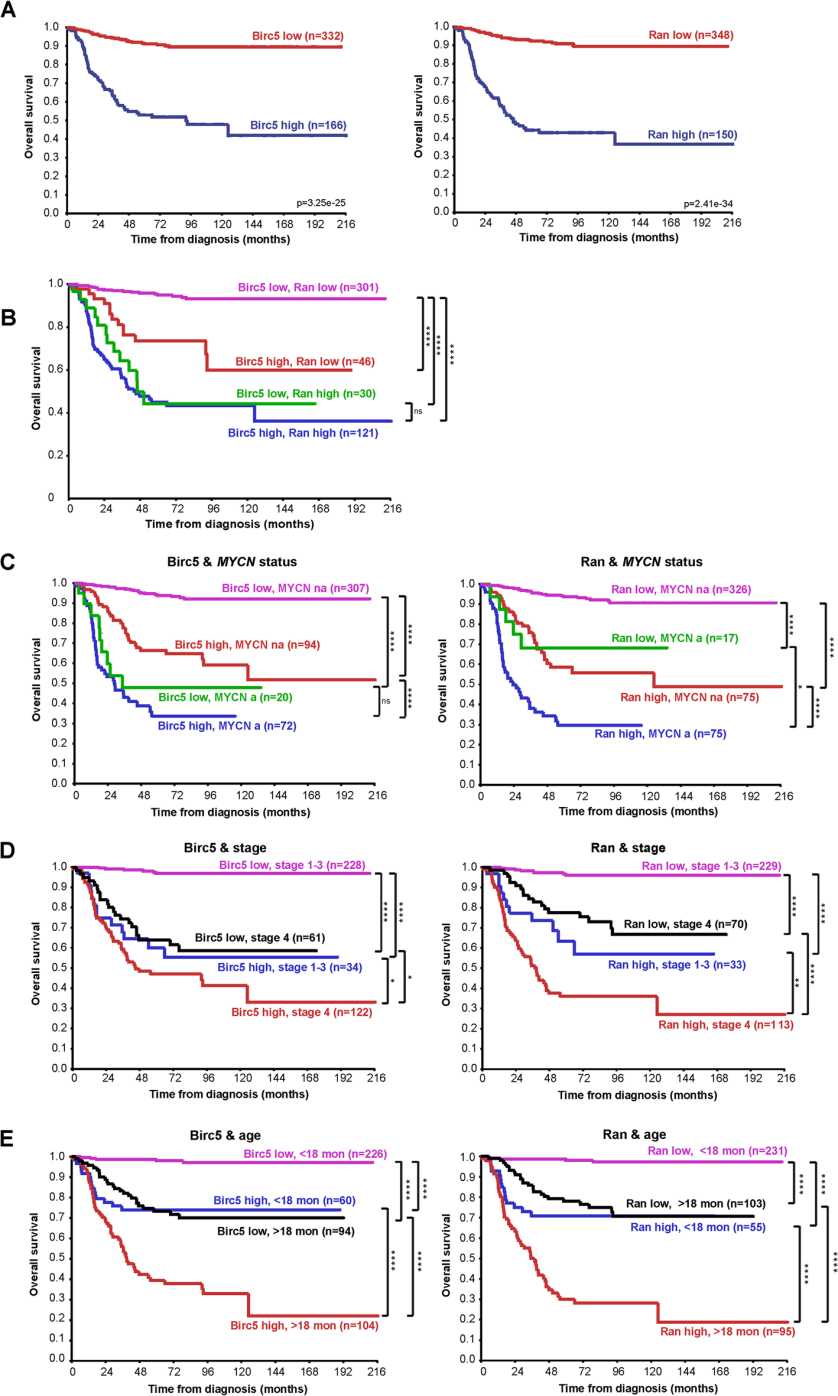
High mRNA expression of Birc5 and Ran is associated with decreased survival of patients. The mRNA expression dataset “SEQC – 498 – RPM – seqcnb1” of 498 NB patients was analyzed in silico using the R2 Genomic analysis and Visualization Platform (http://r2.amc.nl). Transcript levels are categorized into “high” and “low” by maximally selected log-rank statistic. Statistical analysis was performed using the log rank test. **A**) High Birc5 or high Ran expression is associated with poor overall survival of NB patients. Overall survival depending on Birc5 or Ran transcript levels is shown. **B**) High Birc5 and Ran expression is associated with poor overall survival. High expression of Birc5 and Ran is associated with poor prognosis in patients of high and low risk, as determined by **C**) copy number of *MYCN*, **D**) stage, and **E**) age, except for Birc5 when *MYCN *is amplified (**C**)

While increased expression of survivin has previously been linked to poor prognosis in NB [3], the impact of Ran expression, and of survivin and Ran expression combined, was unknown. In silico analysis of a large number of NB patients revealed that both survivin and Ran mRNA expression is associated with markedly decreased patient survival (Fig. 1A, B). Higher expression of survivin and Ran conveys a significant poorer prognosis in patients of high and low risk, as determined by copy number of *MYCN* (Fig. 1C), stage (Fig. 1D) and age (Fig. 1E), except for survivin in tumors with *MYCN* amplification (Fig. 1C). Taken together, these data show the importance of the survivin-Ran nexus in NB.

### LLP-3 controls NB cells in vitro independent of their *MYCN,* p53 and ALK status

**Fig. 2:**
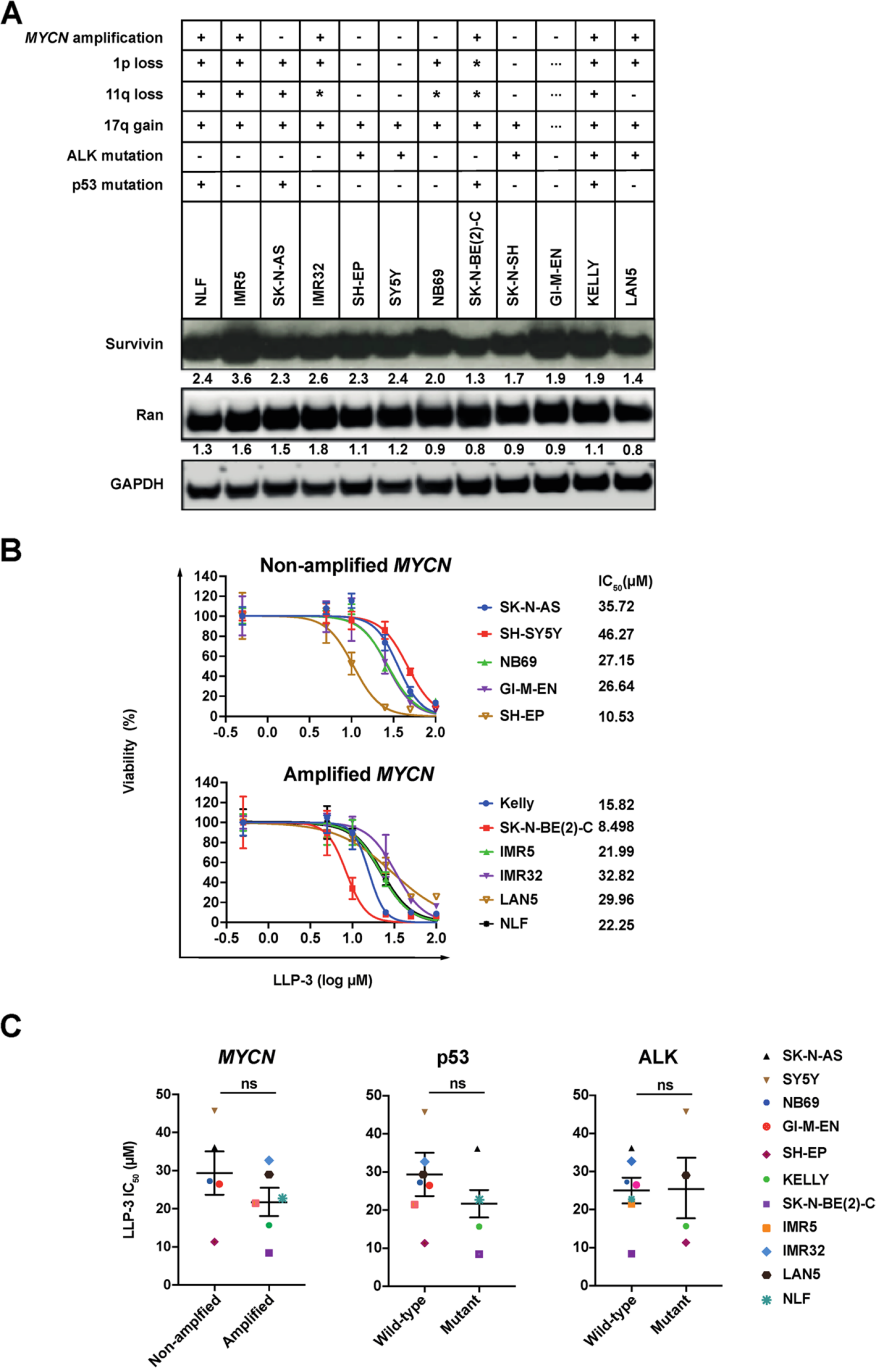
NB cells lines are sensitive to inhibition by LLP-3. **A** Expression of survivin and Ran proteins is not correlated with genetic alterations in NB cells. Western blot analysis of survivin and Ran proteins in a panel of NB cell lines. Presence (+), absence (-), complex changes (*) or insufficient data (...) of important genetic alterations [18–27] are indicated above the blot. Densitometry ratio of protein compared to loading control is shown below bands. Uncropped full-length blots are shown in Suppl. Figure 2. **B** LLP-3 decreases cell viability in NB cells independent of *MYCN* status. Cell viability of *MYCN* non-amplified and of *MYCN*-amplified NB cells after 72 h of exposure to increasing concentrations of LLP-3. Cell viability was measured by MTT and is depicted as percentage of untreated cells. Data are expressed as means±SEM of three independent experiments. IC_50_ were calculated by non-linear regression of log (LLP-3) with fit curves. **C**. LLP-3 decreases cell viability in NB cells independent of *MYCN,* p53 and ALK status*.* Shown are the IC_50_ values of LLP-3 in NB cell lines depending on *MYCN*, p53 and ALK status. Data are expressed as means±SEM of three independent experiments. The unpaired t-test was used for statistical analysis; ns: not significant

**Fig. 3:**
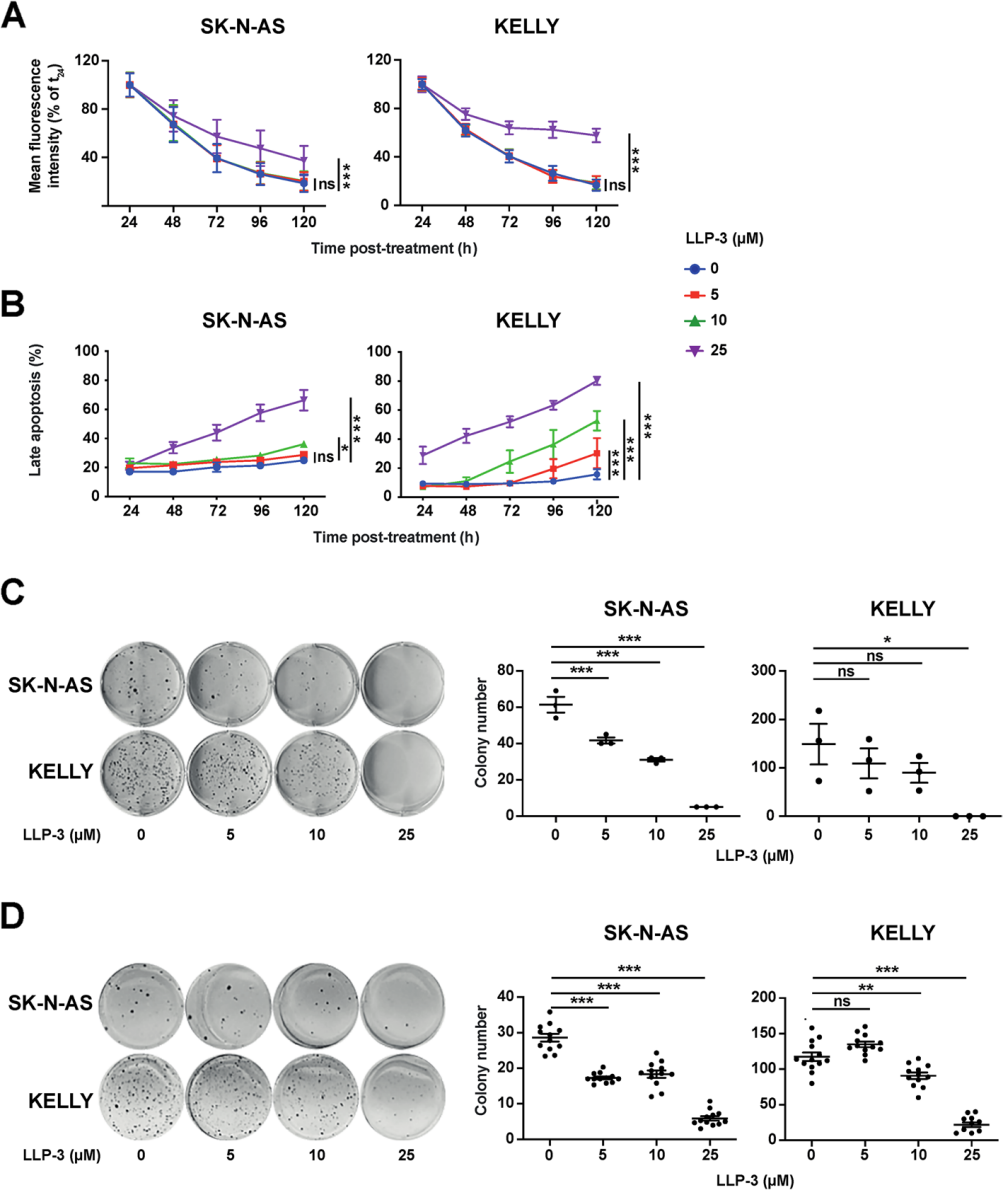
LLP-3 inhibits aggressiveness of SK-N-AS and KELLY NB cell lines. **A** LLP-3 decreases cell proliferation. SK-N-AS and KELLY NB cells were treated with LLP-3 and proliferation was analyzed by flow cytometry. Shown is mean fluorescence intensity as percentage of the signal at 24 h after start of LLP-3 treatment. Data are means±SEM of three independent experiments performed in triplicate. Statistical analysis was performed by two-way ANOVA test. *** *p*<0.001, ns: not significant. **B** LLP-3 induces apoptosis. Late apoptosis was determined by enumerating hypodiploid propidium iodide-stained nuclei. The percentage of apoptotic nuclei of all nuclei is shown. Data are means±SEM of three independent experiments performed in triplicate. Statistical analysis was performed by two-way ANOVA test. *p<0.05, *** *p*<0.001, ns: not significant. **C** LLP-3 inhibits colony formation. Cells growing in plastic dishes were treated with LLP-3 on days 1 and 3 after seeding. After 2–3 weeks cells were stained with crystal violet and colonies were counted. Data are means±SEM of three independent experiments performed in triplicate. Statistical analysis was performed by one-way ANOVA test. *p<0.05, *** *p*<0.001, ns: not significant. **D** LLP-3 inhibits anchorage-independent growth. Cells growing in soft agar were treated with LLP-3 on days 1 and 3 after seeding. After 2–3 weeks cells were stained with MTT and colonies were counted. Data are mean±SEM of twelve replicates of one experiment and repeated two times with similar results. Statistical analysis was performed by one-way ANOVA test. **p<0.01, *** *p*<0.001, ns: not significant

Given the negative impact of increased survivin and Ran on NB prognosis, we set out to investigate the effect of the survivin-Ran inhibitor LLP-3 [15, 16]. First, expression levels of survivin and Ran were determined in a panel of NB cells by immunoblotting. In all 12 NB cell lines survivin and Ran were strongly and consistently expressed, independent of the cell lines’ genetic aberrations (Fig. 2A). Next, we treated a panel of genetically diverse NB cells with LLP-3. All NB cell lines treated were susceptible to LLP-3 (Fig. 2B). Of note, *MYCN*-amplified, p53 mutant and ALK mutant cell lines were as susceptible to LLP-3 as *MYCN* non-amplified, p53 wild-type and ALK wild-type cell lines (Fig. 2B and C). As survivin is essential for mitosis and plays a role in inhibition of apoptosis, we assessed cell proliferation and apoptosis of *MYCN* non-amplified SK-N-AS and *MYCN*-amplified KELLY NB cells in response to LLP-3. Marked inhibition of proliferation, as determined by retention of fluorescent label, and significant apoptosis were evident in both cell lines (Fig. 3A and B). Finally, clonogenic and anchorage-independent growth was markedly and dose-dependently inhibited by LLP-3 in both cell lines (Fig. 3C and D). Taken together, LLP-3 controls NB cells with diverse genetic alterations in vitro*.*

### Survivin and Ran interact both in the nucleus and cytoplasm of NB cells while their expression predominates in the nucleus

**Fig. 4:**
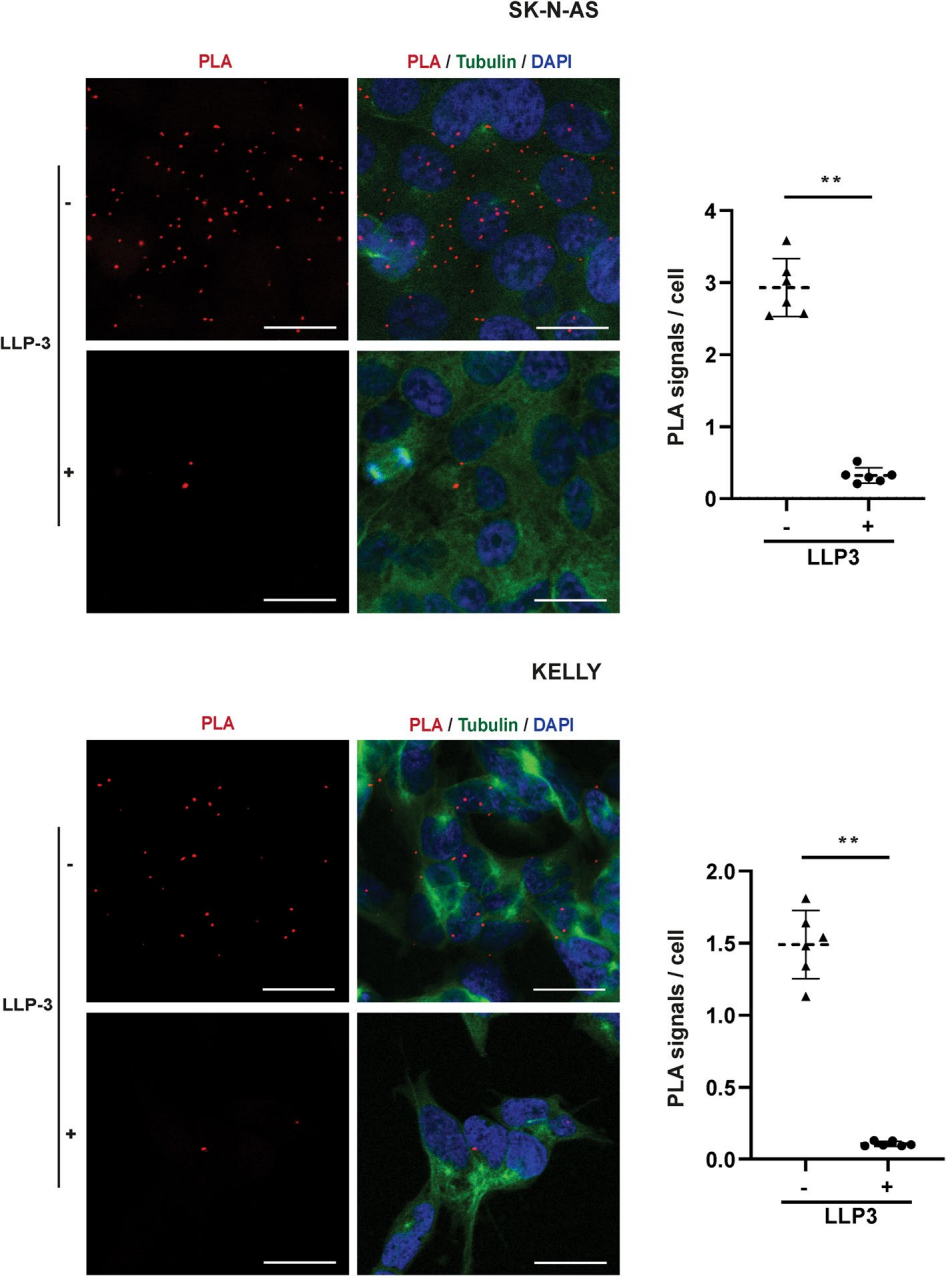
LLP-3 disrupts interaction of survivin with Ran. SK-N-AS and KELLY cells were treated with or without 25 µM LLP-3 for 12 h. Proximity ligation assay (PLA) of survivin-Ran interaction was performed and visualized by confocal microscopy. PLA images were overlayed with tubulin immunofluorescent images and DAPI images outlining the cell borders and nuclei, respectively (left panels). Scale bars correspond to 20 µm. PLA signals were counted in two random visual fields within each of the 3 independent experiments performed (right graphs). Groups were compared using the Mann–Whitney U-test for equality of means. ** indicates *p*<0.01

**Fig. 5:**
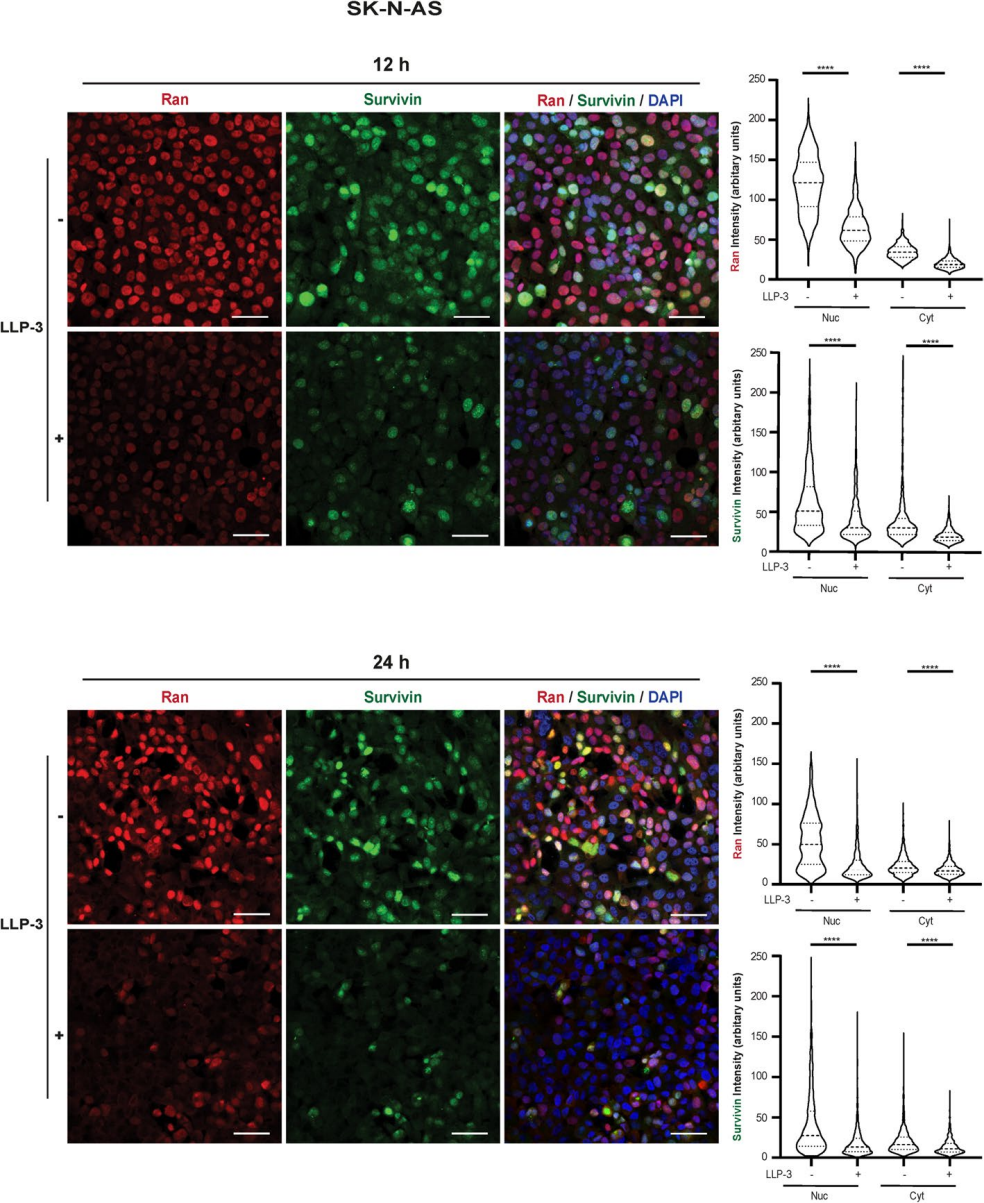
LLP-3 decreases both nuclear and cytoplasmic expression of survivin and Ran in SK-N-AS cells. SK-N-AS cells were treated with or without 25 µM LLP-3 for 12 h (upper panels) and 24 h (lower panels). Confocal microscopy of survivin and Ran immunofluorescence stains and of DAPI stains was performed (left panels, scale bars equal 50 µm). Using these confocal images fluorescence intensity of survivin and Ran in the nuclear (Nuc) and cytoplasmic (Cyt) compartment was determined. Cells of two random visual fields within each of the 3 independent experiments were analyzed. To depict the distribution of single-cell intensities, results are shown as violin plots (upper-right and lower-right panels). Groups were compared using the Mann–Whitney U-test for equality of means. *** indicates *p*<0.0001

As it was unproven whether survivin and Ran interact in NB, which is important for the nuclear-cytoplasmic shuttling of survivin, we performed a proximity-ligation assay. For insight into the subcellular localization of interaction, the assay was visualized by confocal microscopy. Indeed, survivin and Ran interacted in SK-N-AS and KELLY NB cells both in the nucleus and the cytoplasm (Fig. 4). Of note, both survivin and Ran were predominantly located in the nucleus (Fig. 5 and Suppl. Figure 2).

### LLP-3 disrupts survivin-Ran interaction and decreases survivin and Ran in NB cells

Next, we investigated whether LLP-3 inhibited the physical interaction between survivin and Ran. Indeed, LLP-3 markedly disrupted the interaction (Fig. 4). Concomitantly, LLP-3 clearly decreased both the nuclear and cytoplasmic fractions of survivin and Ran in SK-NA-S cells (Fig. 5) and in KELLY cells (Suppl. Figure 2). Taken together, LLP-3 inhibits survivin-Ran interaction and decreases both survivin and Ran proteins in NB cells in a time-dependent manner.

### LLP-3 in NB cells decreases OXPHOS, glycolysis, mitochondrial function and HK activity

**Fig. 6:**
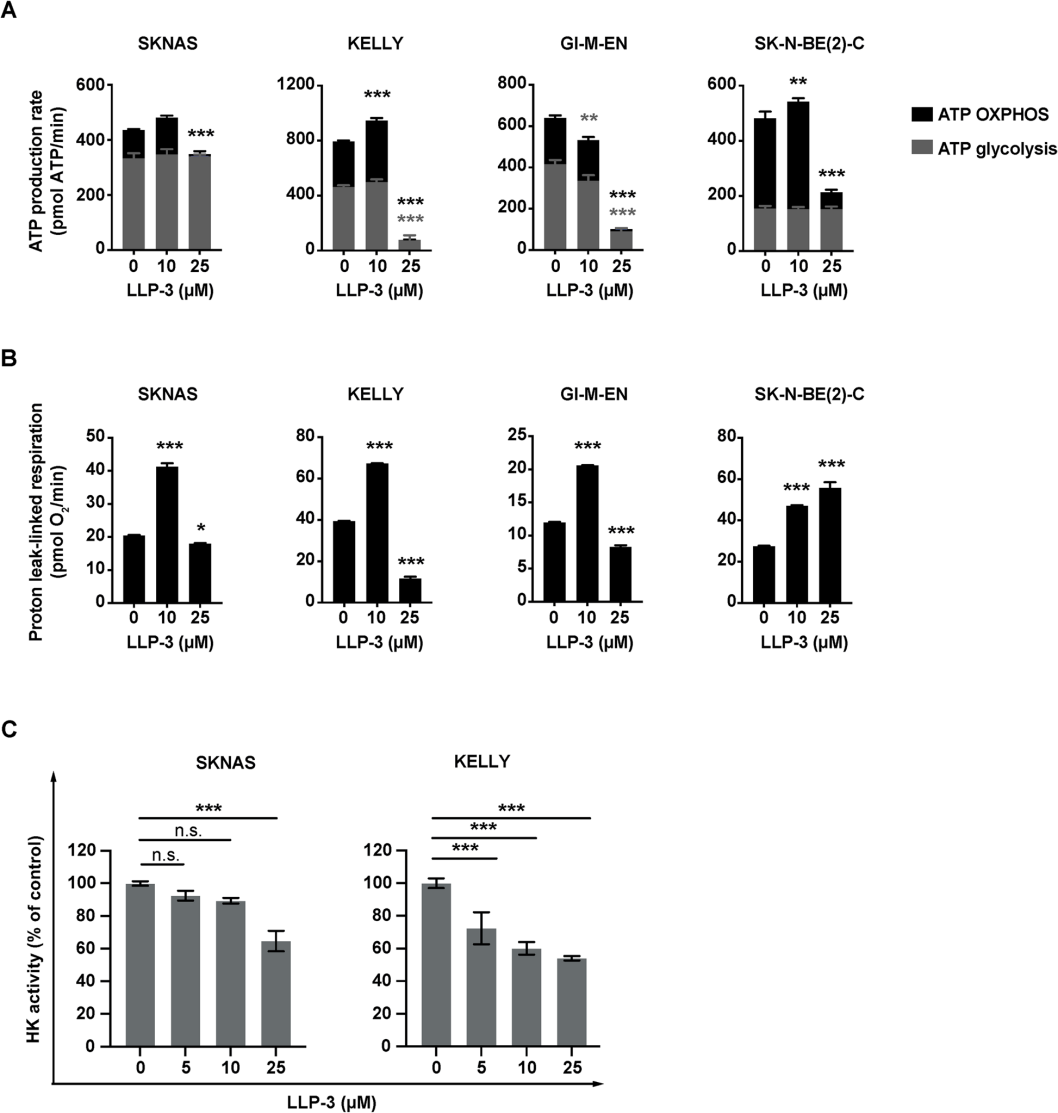
LLP-3 inhibits OXPHOS, aerobic glycolysis, mitochondrial function and HK activity in NB cell lines. Cells were treated with increasing concentrations of LLP-3 for 4 h. ATP production by OXPHOS and glycolysis, and proton leak-linked respiration were measured simultaneously using the Seahorse Extracellular Flux XF96 Analyzer. HK activity was determined by a colorimetric assay. **A** LLP-3 inhibits OXPHOS and glycolysis at high concentration. ATP production rates from OXPHOS and glycolysis are shown as means ± SEM from 5 independent experiments performed in quintuplicates. Data were analyzed by two-way ANOVA test. **p<0.01, ***p<0.001. **B** LLP-3 causes mitochondrial dysfunction at low concentration. Proton leak-linked respiration data are depicted as means ± SEM from 5 independent experiments performed in quintuplicates. Data were analyzed by two-way ANOVA test. *p<0.05, *** *p*<0.001. **C** LLP-3 inhibits HK activity. Data are shown as means±SEM from 3 (SK-N-AS) and 2 (KELLY) independent experiments performed in triplicates. Data were analyzed by one-way ANOVA test. *** *p*<0.001; ns, not significant

Since LLP-3 decreased survivin in NB cells and because survivin has been implicated in OXPHOS and aerobic glycolysis, we investigated the effect of LLP-3 in energy metabolism of NB cells. In cancer cells, including NB cells, a fraction of survivin is found in the mitochondrial matrix. Both promotion of OXPHOS, and decrease of OXPHOS with increase of glycolysis have been reported in relation to mitochondrial-bound survivin [7–9]. We therefore reasoned that LLP-3 may modulate OXPHOS and glycolysis. *MYCN* non-amplified NB cells (SK-N-AS and GI-M-EN) and *MYCN*-amplified NB cells (KELLY and SK-N-BE(2)-C) were subjected to extracellular flux-based analyses of OXPHOS, glycolysis and proton-linked respiration. Oxidative ATP production rates increased at low LLP-3 concentration in most cell lines (Fig. 6A) while markedly decreasing at high concentration in all, indicating inhibition of OXPHOS at high LLP-3 concentration (Fig. 6A). Glycolytic ATP production rates were reduced in KELLY and GI-M-EN cells, while not inhibited in SK-N-AS and SK-N-BE(2)-C cells (Fig. 6A). Proton leak respiration increased at low concentration of LLP-3 in all cell lines, indicating mitochondrial dysfunction (Fig. 6B). Thus, LLP-3 in NB cells causes mitochondrial dysfunction at lower concentration, and marked inhibition of OXPHOS and glycolysis at higher concentration.

As LLP-3 inhibited energy metabolism, we investigated whether LLP-3 inhibits activity of HK2, a gatekeeper of energy metabolism [28]. Activity of HK rapidly decreased in response to LLP-3 (Fig. 6C). This response occurred already at lower LLP-3 concentrations in KELLY cells compared to SK-N-AS cells. No consistent changes in the expression of other key enzymes of OXPHOS and glycolysis were observed (Suppl. Figures 3 and 4).

Because the transcriptional activator HIF-1α has been described to affect both OXPHOS and glycolysis [29], and is upregulated in high-risk NB [30], the role of HIF-1α was explored. In SK-N-AS cells LLP-3 treatment did not diminish HIF-1α and in KELLY cells only an insignificant decrease occurred (Suppl. Figures 5A and 6A). While stabilization of HIF-1α shifted energy metabolism toward glycolysis, this did not alter the effect of LLP-3 on OXPHOS and glycolysis (Suppl. Figure 5B). Furthermore, no physical interaction between survivin and HIF-1α - that may be a target for LLP-3 - was detected (Suppl. Figures 5C and 6B.) Thus, LLP-3-induced decrease of OXPHOS and glycolysis is independent of HIF-1α.

Collectively, these data show that LLP-3 in NB cells decreases OXPHOS, glycolysis, mitochondrial function and HK activity.

## Discussion

Given the poor prognosis of high-risk NB, novel therapeutic approaches are needed. A promising approach is to target survivin in NB. We now provide evidence that the survivin-Ran inhibitor LLP-3 [15–17] controls NB cells in vitro, associated with impaired flexibility of energy metabolism due to inhibition of both OXPHOS and glycolysis.

High survivin and Ran transcript levels in NB tumors were strongly associated with decreased patient survival. While the strong negative impact of increased survivin expression is known, the even stronger impact of Ran is remarkable. Furthermore, the results do not contradict the notion that expression of Ran and survivin may be independent prognostic factors in low-risk NB.

The patient data indicated that targeting survivin and Ran simultaneously might be a rational therapeutic approach to NB. Indeed, LLP-3 decreased viability, induced apoptosis and inhibited clonogenic and anchorage-independent growth in a panel of NB cell lines. Clonogenic and anchorage-independent growth was inhibited at lower doses that did not affect proliferation and apoptosis, suggesting particular efficacy of LLP-3 on cellular functions important for tumor spread. Of note, also *MYCN*-amplified, ALK-mutated and p53-mutated NB cells were susceptible to LLP-3. The latter is in line with glioma stem cells [15] and colorectal cancer cell lines that are sensitive to LLP-3 in the presence of p53 mutations [16]. Along this line, the efficacy of YM155, which decreases transcription of survivin, appears not to be influenced by the function of p53 in NB [10].

Survivin and Ran were found bound to each other both in the nucleus and the cytoplasm while expression of survivin and Ran predominated in the nucleus. When considering any prognostic impact of either nuclear or cytoplasmic localization of survivin in NB, it should be noted that this impact has been described to be heterogeneous in other cancer types [4]. LLP-3 effectively disrupted survivin-Ran interaction associated not only with a decrease of survivin but also of Ran. While LLP-3-induced decrease of survivin has been described [16], the simultaneous association of decreased survivin and Ran is a novel finding. It supports the notion of a two-pronged effect of LLP-3 on survivin and Ran protein levels. Along this line, Ran is involved in the nucleocytoplasmic transport of various proteins important for cell homeostasis [31].

As LLP-3 markedly decreased survivin levels, the impact of LLP-3 on the metabolic function of survivin was investigated. Indeed, LLP-3 caused proton leak indicative of mitochondrial dysfunction at lower concentration, and marked inhibition of OXPHOS and glycolysis at higher concentration. Of note, inhibition of glycolytic ATP production was cell-type specific, indicating differences in glycolytic enzyme expression. Our data indicate that LLP-3 severely impairs the flexibility of energy metabolism of the NB cells by preventing them to switch between OXPHOS and glycolysis if needed. Metabolic flexibility is crucial for survival of cancer cells under metabolic challenging conditions such as hypoxia or metastasis.

A conceivable mechanism of how LLP-3 could have attenuated OXPHOS and glycolysis was to impact on HIF-1α, the transcription factor pivotal for both modes of energy metabolism [29]. However, a series of experiments refuted this hypothesis. HIF-1α did not decrease consistently and markedly by LLP-3 and did not alter the effect of LLP-3 on OXPHOS and glycolysis. Furthermore, no physical interaction between HIF-1α and survivin was detected, thus precluding that LLP-3 could disrupt such an interaction, subsequently decreasing stability of HIF-1α. LLP-3 also did not diminish expression of GLUT1, PKM2, PDH, PDK1 and LDHA, other key enzymes in OXPHOS or aerobic glycolysis at an early time point, although it remains possible that changes in expression occur later.

Thus, other mechanisms may be operative in LLP-3-induced inhibition of energy metabolism and therefore metabolic flexibility. Hexokinase 2 (HK2), preferentially expressed in cancers, is bound to the outer mitochondrial membrane (OMM), where it has privileged access to ATP generated in the mitochondria, thus facilitating phosphorylation of glucose, crucial for both OXPHOS and aerobic glycolysis [28]. In addition, HK2 is known to inhibit binding of pro-apoptotic factors to the OMM and to decrease mitochondrial ROS production [28]. We observed decreased activity of HK in response to LLP-3, which may have contributed to the decrease of OXPHOS and aerobic glycolysis. It remains to be elucidated how the inhibitory effect of LLP-3 on HK is mediated. Furthermore, LLP-3 may inhibit the pool of survivin known to be present at the mitochondrial membrane and to regulate OXPHOS and glycolysis [7–9]. This would support the notion that mitochondrial survivin enhances OXPHOS [8] rather than inhibiting it while enhancing aerobic glycolysis [7]. It remains to be investigated whether LLP-3 indeed inhibits the mitochondrial pool of survivin and how this would impact on mitochondrial integrity [7, 9], OXPHOS Complex I [8] or II [7], and glycolysis [7, 9].

In summary, LLP-3 inhibits interaction and levels of survivin and Ran in NB cells. It effectively kills NB cells with diverse genetic alterations, associated with inhibition of OXPHOS, aerobic glycolysis, mitochondrial function and HK activity, in sum impairing flexibility of energy metabolism. LLP-3 is therefore a promising novel drug for NB therapy that warrants further studies.

### Supplementary Information

**Additional file 1: Suppl. Fig. 1.** Uncropped full-length Western blots of Fig. 2A. **Suppl. Fig 2.** LLP-3 decreases both nuclear and cytoplasmic expression of survivin and Ran in KELLY cells by 24 h. **Suppl. Fig. 3.** LLP-3 does not consistently alter expression of GLUT1, HK2, PKM2, LDHA, PDK1 and PDHA. **Suppl. Fig. 4.** Uncropped full-length Western blots of Suppl. Fig. 3. **Suppl. Fig. 5.** HIF-1α protein does not alter the effect of LLP-3 on NB cell lines. **Suppl. Fig. 6.** Uncropped full-length Western blots of Suppl. Fig. 5A (A) and Suppl. Fig. 5C (B).

## Data Availability

The datasets used and/or analyzed during the current study are available from the corresponding author on reasonable request.

## Abbreviations

ALK: Anaplastic lymphoma kinase
BIRC5: Baculoviral inhibitor of apoptosis repeat-containing 5
Co-IP: Co-immunoprecipitation
CoCl_2_: Cobalt (II) chloride
ECAR: Extracellular acidification rate
GLUT1: Glucose transporter 1
HIF-1α: Hypoxia-inducible factor 1-alpha
HK2: Hexokinase 2
HK activity: Hexokinase activity
IC50: Half maximal inhibitory concentration
LDHA: Lactate Dehydrogenase A
MTT: 3-(4,5-Dimethylthiazol-2-yl)-2,5-diphenyl-2H-tetrazolium bromide (MTT) assay
MYCN: V-myc avian myelocytomatosis viral oncogene neuroblastoma derived homolog
OCR: Oxygen consumption rate
OMM: Outer mitochondrial membrane
OXPHOS: Oxidative phosphorylation
NB: Neuroblastoma
PDHA: Pyruvate Dehydrogenase alpha
PDK1: Pyruvate Dehydrogenase Kinase 1
PKM2: Pyruvate kinase M2
Ran: Ras-related nuclear protein
