# Supplementary material for: The survivin-ran inhibitor LLP-3 decreases oxidative phosphorylation, glycolysis and growth of neuroblastoma cells
Source: BMC Cancer. 2023 Nov 25;23:1148. doi: 10.1186/s12885-023-11635-2 (PMC10676583; doi:10.1186/s12885-023-11635-2)
Supplement: Supplementary file 1 — Additional file 1: Suppl. Fig. 1. Uncropped full-length Western blots of Fig. 2A. Suppl. Fig 2. LLP-3 decreases both nuclear and cytoplasmic expression of survivin and Ran in KELLY cells by 24 h. Suppl. Fig. 3. LLP-3 does not consistently alter expression of GLUT1, HK2, PKM2, LDHA, PDK1 and PDHA. Suppl. Fig. 4. Uncropped full-length Western blots of Suppl. Fig. 3. Suppl. Fig. 5. HIF-1α protein does not alter the effect of LLP-3 on NB cell lines. Suppl. Fig. 6. Uncropped full-length Western blots of Suppl. Fig. 5A (A) and Suppl. Fig. 5C (B). [file 12885_2023_11635_MOESM1_ESM.docx]

**Supplemental information**

**Suppl. Fig. 1. Uncropped full-length Western blots of Fig. 2A**.

**Suppl. Fig 2. LLP-3 decreases both nuclear and cytoplasmic expression of survivin and Ran in KELLY cells by 24 h.** KELLY cells were treated with or without 25 µM LLP-3 for 12 h (upper panels) and 24 h (lower panels). Confocal microscopy of survivin and RAN immunofluorescence stains and of DAPI stains was performed (left panels, scale bars equal 50 µm). Using these confocal images fluorescence intensity of survivin and RAN in the nuclear (Nuc) and cytoplasmic (Cyt) compartment was determined. Cells of two random visual fields within each of the 3 independent experiments were analyzed. To depict the distribution of single-cell intensities, results are shown as violin plots (upper-right and lower-right panels). Groups were compared using the Mann-Whitney U-test for equality of means. **** indicates p < 0.0001, ns not significant.

**
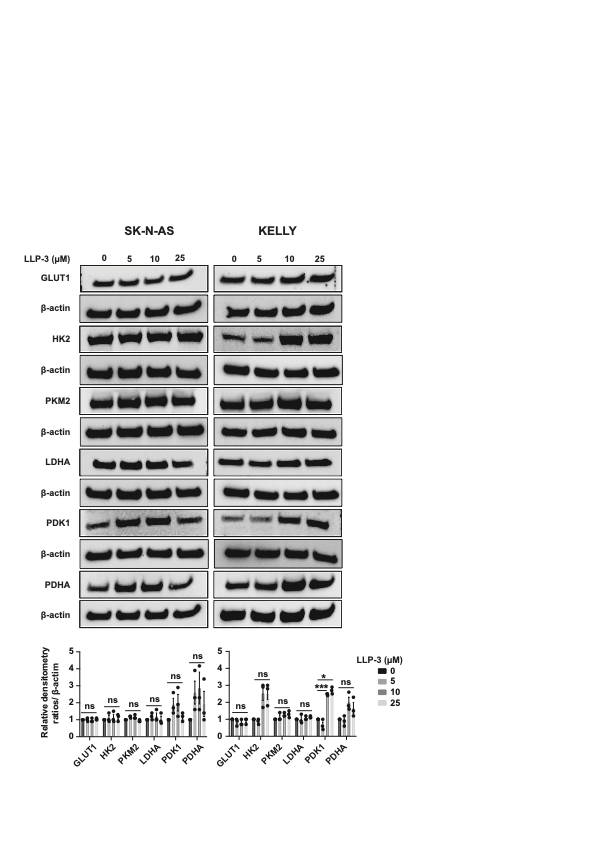
**

**Suppl. Fig. 3. LLP-3 does not consistently alter expression of GLUT1, HK2, PKM2, LDHA, PDK1 and PDHA**. Cells were treated with LLP-3 for 4 h. Western blots are shown in the upper panel, β-actin was used as loading control**.** Densitometric analysis is shown in the lower panel. Data are depicted as means ± SEM of three independent experiments performed in quintuplicates. Two-way ANOVA test was applied. *** *p* < 0.001; ns, not significant. Uncropped full-length blots are shown in Suppl. Fig. 4.

**Suppl. Fig. 4. Uncropped full-length Western blots of Suppl. Fig. 3**


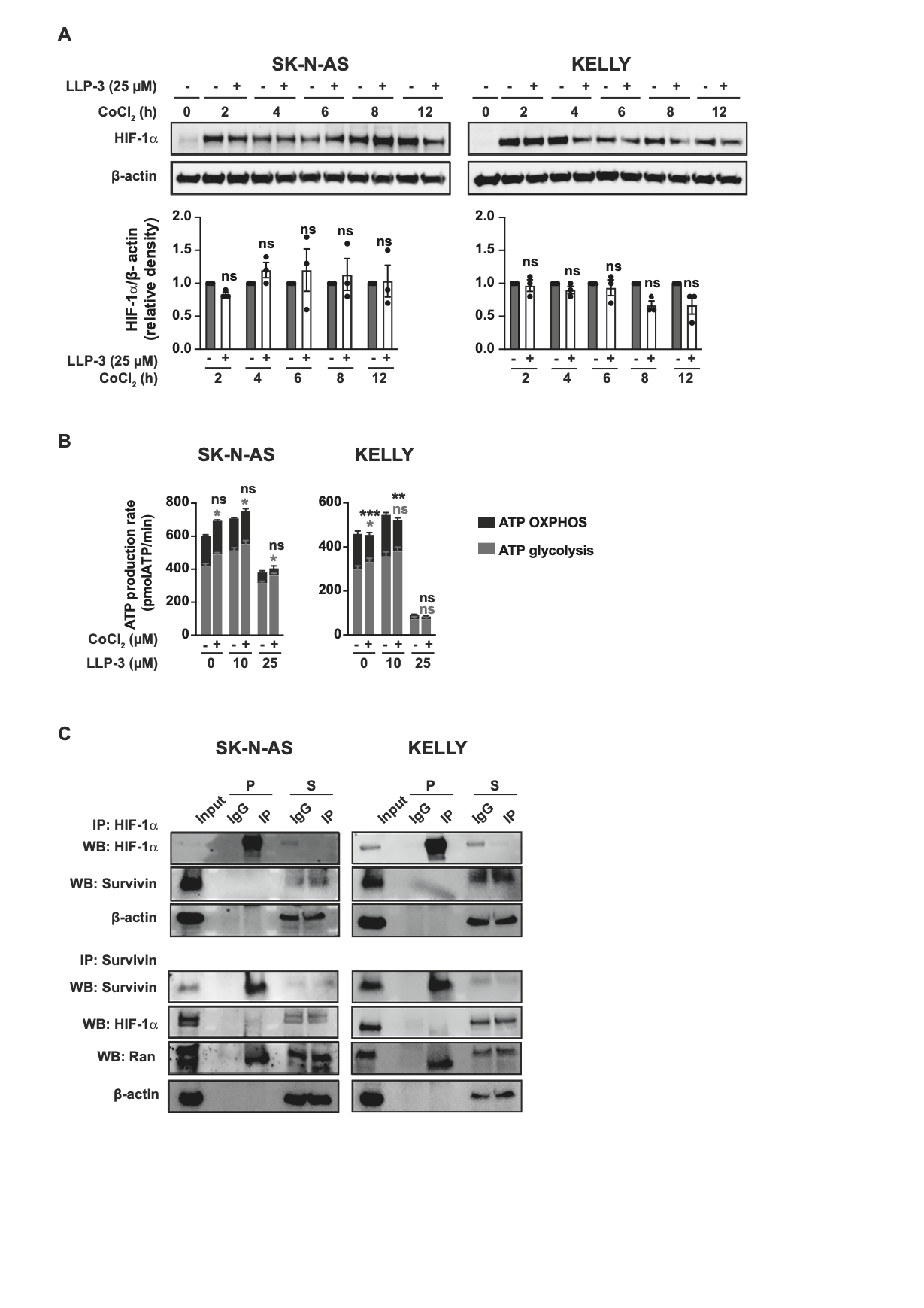


**Suppl. Fig. 5. HIF-1α protein does not alter the effect of LLP-3 on NB cell lines.** **(A)** **LLP-3 does not decrease HIF-1α expression.** Cells were treated with LLP-3 for increasing time. At the time points indicated cells were incubated for 2 h with medium containing CoCl_2_ and no LLP-3. Data are means ± SEM of three independent experiments. Two-way ANOVA test was performed. ns; not significant. Uncropped full-length blots are shown in Suppl. Fig. 6A. **(B) Stabilization of HIF-1α does not protect against the inhibitory effect of LLP-3 on OXPHOS and glycolysis**. Cells were treated with LLP-3 for 4 h and then incubated for 2 h with medium containing CoCl_2_ and no LLP-3**.** ATP production rates from OXPHOS (upper panel) and glycolysis (lower panel), as determined by the Seahorse Extracellular Flux XF96 Analyzer, are shown. Data are means ± SEM of three independent experiments performed in quintuplicates. Two-way ANOVA test was performed. **p* < 0.05, ** *p* < 0.01; ns, not significant. **(C)** **No interaction between survivin and HIF-1α proteins.** Co-immunoprecipitations of HIF-1α with survivin and of survivin with HIF-1α were performed. P, pellet; S, supernatant; IP, immunoprecipitation; IgG, unspecific IgG antibody. β-actin was used as loading control. Uncropped full-length blots are shown in Suppl. Fig. 6B. Two experiments were performed, with similar results.

**Suppl. Fig. 6**. **Uncropped full-length Western blots of Suppl. Fig. 5A (A) and Suppl. Fig. 5C (B).**
